# Evaluating Fundoscopy as a Screening Tool for Optic Nerve Atrophy in Multiple Sclerosis: An Optical Coherence Tomography (OCT) Comparative Study

**DOI:** 10.3390/jcm14072166

**Published:** 2025-03-22

**Authors:** Vlad Constantin Donica, Ciprian Danielescu, Anisia Iuliana Alexa, Irina Andreea Pavel, Alexandra Lori Donica, Cristina Grosu, Călina Anda Sandu, Camelia Margareta Bogdănici

**Affiliations:** 1 Department of Ophthalmology, Faculty of Medicine, University of Medicine and Pharmacy “Grigore T. Popa”, University Street No. 16, 700115 Iasi, Romania; vlad-constantin.donica@umfiasi.ro (V.C.D.); camelia.bogdanici@umfiasi.ro (C.M.B.); 2 Department of Rheumatology, Faculty of Medicine, University of Medicine and Pharmacy “Grigore T. Popa”, University Street No. 16, 700115 Iasi, Romania; 3 Department of Neurology, Faculty of Medicine, University of Medicine and Pharmacy “Grigore T. Popa”, University Street No. 16, 700115 Iasi, Romania; fcristina_ro@yahoo.com

**Keywords:** multiple sclerosis, OCT, fundoscopy, optic disc atrophy

## Abstract

**Background:** Multiple sclerosis (MS) is a chronic, immune-mediated disorder of the central nervous system (CNS), characterized by inflammation, demyelination, and neurodegeneration, resulting in the disruption of axonal signal conduction. Optic neuritis (ON) occurs in over 70% of MS cases, highlighting the involvement of the optic nerve in the progression of the disease. Optic nerve atrophy secondary to the inflammatory episode can be observed during fundoscopy as pallor in the temporal quadrant or of the entire optic disc. Our study aims to evaluate the diagnostic capacity of fundus ophthalmoscopy when compared with the temporal thickness of the pRNFL (peripapillary retinal nerve fiber layer) measured using optical coherence tomography (OCT). **Methods**: We analyzed 88 eyes from 44 relapsing remitting MS patients using fundus photography (FP) and OCT optic disc measurements, correlating the temporal pallor of the optic disc seen in fundus photographs (FPs) with structural parameters obtained using OCT. **Results**: Our analysis revealed the significant capacity of optic disc pallor grading using FPs in MS patients in order to discriminate between normal and quadrants with pallor (*p* = 0.006) or strong pallor (*p* = 0.003) and between ones with light pallor and moderate pallor (*p* = 0.002) or strong pallor (*p* = 0.001), while being unable to clearly differentiate between normal quadrants and ones with light pallor (*p* = 0.608) or between pallor and strong pallor (*p* = 0.33). **Conclusions**: Fundoscopy and FP are useful screening tools in evaluating optic nerve atrophy in MS patients that could be used to assess neurodegeneration because of their universal availability. With the proposed inclusion of the optic disc as the fifth part of the CNS, the optic nerve will benefit from multiple exploratory techniques in order to increase the understanding of disease progression and patient quality of life.

## 1. Introduction

Multiple sclerosis (MS) is a chronic, immune-mediated, demyelinating disease of the central nervous system (CNS), characterized pathologically by the perivascular infiltration of autoreactive T and B lymphocytes, macrophage-mediated myelin degradation, oligodendrocyte loss, axonal transection, and astrocytic gliosis, leading to the formation of demyelinated plaques in both white and gray matter. The disease exhibits spatiotemporal dissemination of lesions and is driven by a complex interplay of genetic susceptibility and environmental factors. MS is marked by neuroinflammatory episodes and progressive neurodegeneration, resulting in impaired saltatory conduction, axonal dysfunction, and irreversible neurological disability [1].

Optic neuritis (ON) is the first manifestation of MS in 20% of cases, and over 70% of patients will experience an episode during the course of the disease. Common symptoms include decreased visual acuity, periocular pain triggered by eye movement, dyschromatopsia, reduced contrast sensitivity, and visual field defects [2]. The damage sustained in the optic pathway is a result of both acute CNS injury and trans-synaptic neurodegeneration. This reaction occurs behind the lamina cribrosa, which explains the inflammatory status of the optic nerve during ON [3,4].

After the resolution of acute ON, most patients exhibit some degree of peripapillary retinal nerve fiber layer (pRNFL) loss, detectable on fundoscopic examination as pallor of the optic disc [5]. For many years, optic disc atrophy was mainly assessed by the pallor level of the temporal quadrant of the optic disc during fundus ophthalmoscopy.

The proposed inclusion of the optic nerve as a fifth region of the central nervous system could lead to the earlier and more accurate diagnosis of MS patients and, therefore, to an increase in the interest in optic disc alterations [6,7].

The development of OCT-Angiography (OCT-A) added new parameters that can be used to evaluate and quantify the severity of optic disc degeneration, and while OCT is not included in the current 2017 McDonald criteria for MS diagnosis, it can be used as a complementary tool in MS evaluation and in differentiating between optic neuropathies [8,9]. Despite the availability of OCT worldwide, MS patients do not always benefit from proper ophthalmological referral or long-term follow-up to analyze changes in the optic disc and retinal physiology. Therefore, many physicians still evaluate the level of optic disc atrophy based on the aspect of fundoscopy prior to conducting an OCT evaluation.

The use of OCT in detecting neurodegeneration of the optic disc and retina has been proven to have increased sensitivity and repeatability [10]. The pRNFL and the ganglion cell layer (GCL) have been the most recognized altered parameters in MS patients, establishing as imagistic biomarkers in disease diagnosis and activity evaluation [11,12]. Although pRNFL assessment was among the first accessible tools for evaluating the optic nerve, contradictions remain regarding the results, which may arise from the small number of subjects assessed, the use of different imaging and analysis technologies, segmentation errors, or the progression of the pathology into new entities that were not defined at the time of the initial examination. One of the limitations of the pRNFL assessment is the presence of peripapillary edema at the onset of ON. Axonal lesions cannot be evaluated until the resolution of the inflammatory episode, which causes thickening of the pRNFL as measured by OCT [13]. The degree of pRNFL atrophy present one month after the inflammatory episode provides significant information regarding visual prognosis, with thinning after one month suggesting a greater degree of atrophy at six months [14]. Thus, in the eyes of MS patients with a history of an ON episode, the evaluation of structural parameters in the macular area provides superior information about the current degree of nerve degeneration, with thinning observed approximately 1–2 months after the onset of the inflammatory episode [15].

Retinal imaging has been utilized in medicine since 1886, initially being used as an office-based, technician-dependent method for objectifying ophthalmological diseases. It has since evolved hand in hand with technology, becoming a portable, efficient, and low-cost imaging modality, used in order to perform screening and remote diagnosis and, therefore, becoming highly suitable for telemedicine [16].

Our study aims to evaluate the diagnostic ability of fundus photography evaluations to distinguish between levels of optic nerve atrophy by comparing the results with the ability of OCT to quantify the temporal thickness of the pRNFL. While there are studies that used a color grading system for the analysis of optic disc images, the authors used computer imaging software to objectively assess hue and saturation and compared it with the subjective grading from other examiners [17].

## 2. Materials and Methods

The study design and protocol were conducted in accordance with the tenets of the Declaration of Helsinki for research involving human subjects and approved by the Ethics Committee of “Grigore T. Popa” University of Medicine and Pharmacy Iasi, Romania (No. 331/approval date on 12 July 2023). Written informed consent was obtained prior to patient evaluation.

We performed a cross-sectional analysis of 88 eyes from 44 relapsing remitting MS patients. The inclusion criteria included a positive history of relapsing remitting MS, more than 3 months after an ON episode, good FP image quality, and good OCT segmentation. The exclusion criteria included the coexistence of other optic neuropathies, a recent episode of ON, ophthalmological disease that causes low image resolution, or a decreased OCT quality index. The 3 months after the ON episode were necessary in order to allow the peripapillar edema to resolve and observe the remaining optic nerve damage.

OCT and color fundus photographs (FPs) of the optic disc were obtained by an experienced examinator using the TRITON swept-source OCT device (DRI OCT Triton, Topcon, Tokyo, Japan), ensuring the same light settings and parameters. The OCT disc scans were reviewed to ensure correct segmentation and high image quality. Layer segmentation and measurements were performed automatically by the integrated Triton software.

The optic disc FPs were evaluated by two ophthalmology physicians with neuro-ophthalmological expertise and graded based on the temporal pallor level compared with other sectors of the optic disc. The normal aspect had an intense orange appearance, while the light-pallor group had a lighter orange appearance when compared with the other quadrants of the disc rim. The pale group had a yellow aspect, when compared with the rest of the disc, while strong-pallor discs had a white, waxy color (Table 1).

**Figure 1:**
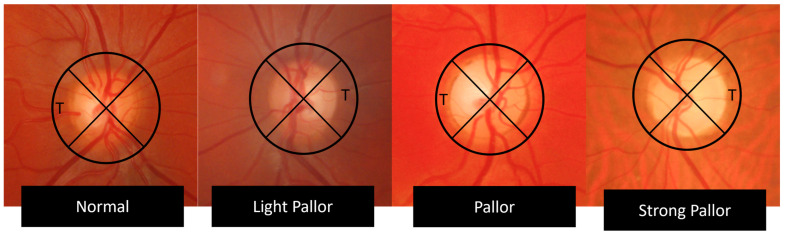
Temporal pallor evaluation using FP. T; temporal quadrant.

Figure 1 illustrates how the examiners graded temporal optic disc pallor from normal to light pallor, moderate pallor, and strong pallor.

**Figure A1:**
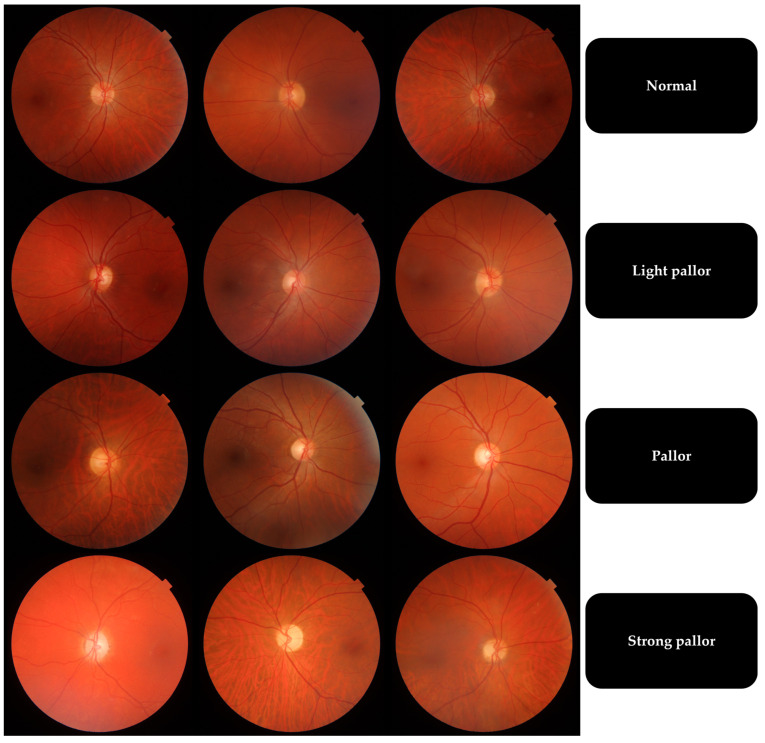
Examples of FP temporal pallor grading.

A third examiner was asked to evaluate cases in which there was disagreement. Additional examples of the analyzed images can be viewed in Figure A1 at the end of this manuscript. We compared the examiner analysis from the FP with the OCT temporal pRNFL values in order to quantify pallor level with nerve layer thickness.

### Statistical Analysis

Statistical analysis was performed using SPSS statistical package, version 26.0. Cohen’s κ was run to determine the agreement relationship between the two examiners. A Kruskal–Wallis test was conducted to determine whether there were differences between gradings that differed in pallor level in the fundus photography: the “Normal” (*n* = 29), “Light pallor” (*n* = 26), “Pallor” (*n* = 24), and “Strong pallor” (*n* = 9) and OCT temporal thickness values. All the data are presented as a mean ± SD (standard deviation) unless otherwise stated. The distribution of the grading was similar for all the groups, as assessed by visual inspection of a boxplot. The median scores were statistically significantly different between the different aspects of disc pallor, χ^2^(3) = 18.736, *p* < 0.0005. Subsequently, pairwise comparisons were performed using Dunn’s (1964) procedure with a Bonferroni correction for multiple comparisons. The adjusted *p*-values are presented.

## 3. Results

Our analysis included 88 eyes from 44 RRMS patients. The average age was 37.5 ± 1.98 years, with the youngest patient having been 18 years of age at image capture, while the oldest was 56 years of age. Regarding the gender incidence, we observed a 2.67 female-to-male ratio.

**Figure 2:**
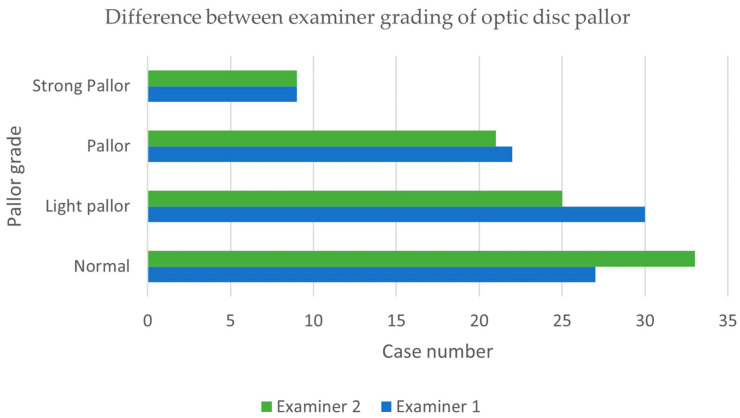
Difference between examiner grades regarding optic disc pallor.

Examiner 1 graded 27 discs as normal, 30 as light pallor, 22 as pallor, and 9 as strong pallor, while examiner 2 observed 33 normal discs, 25 with light pallor, 21 with pallor, and 9 with strong pallor. There was a good level of agreement between the examiners, k = 0.76 (95%CI, 0.65 to 0.87), *p* < 0.0005. The agreement level between examiners can be observed in Figure 2.

**Figure 3:**
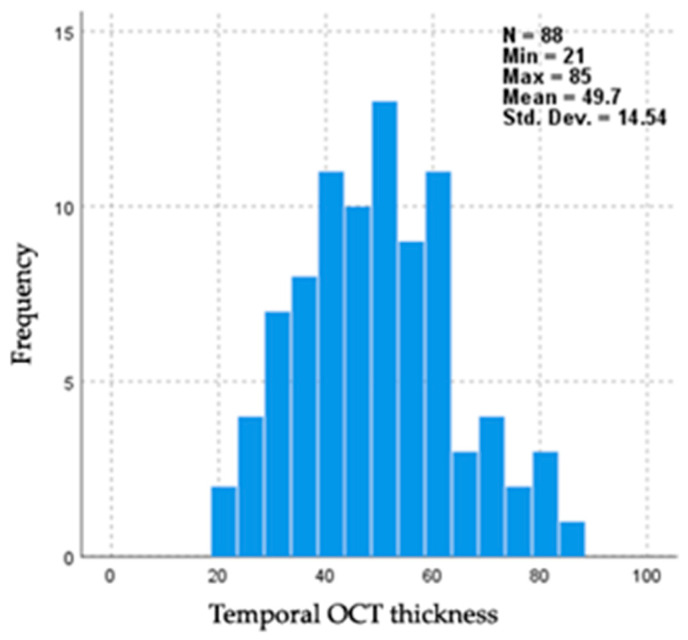
Temporal pRNFL thickness value frequency.

A third examiner was asked to grade and resolve the disagreement between the initial graders. After resolving the disagreements, the final analysis graded 29 as normal, 26 with light pallor, 24 as pale, and 9 with strong pallor (Figure 3).

**Figure 4:**
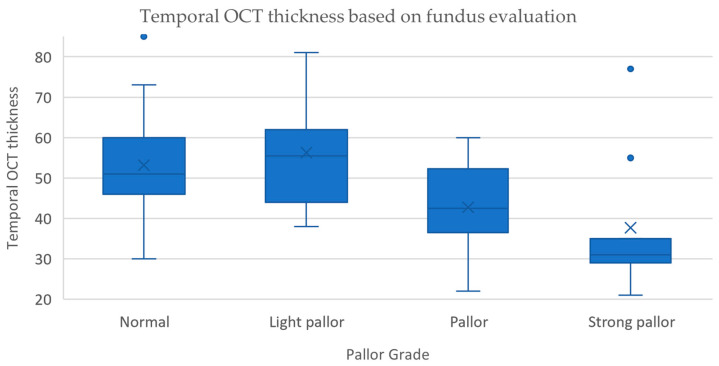
Correlation between examinator grade and temporal pRNFL thickness.

The normal discs correlated on OCT with a mean temporal thickness of the pRNFL of 53.17 ± 12.49 µm, those with light pallor with a thickness of 56.27 ± 13.67 µm, the pale with a mean thickness of 42.75 ± 11.34 µm, while those with strong pallor correlated with a thickness of 37.67 ± 17.48 µm (Table 2). A schematic representation can be seen in Figure 4.

The post hoc analysis revealed statistically significant differences in the median pRNFL thickness and examinator scores between the normal optic discs and ones with pallor (*p* = 0.006) and strong pallor (*p* = 0.003), between light pallor and ones with pallor (*p* = 0.002) and strong pallor (*p* = 0.001), while no significant difference could be identified between the normal and light pallor (*p* = 0.608) and the pallor and strong pallor (*p* = 0.33) group combination comparisons (Table 3).

## 4. Discussion

The use of imaging in retinal disorders has been widely accepted as a valid technique for diagnosing retinal and optic disc diseases in daily clinical practice. It can enhance the quality of the examination by providing patient images in different moments, being able to detect recurrence and degeneration [18]. Furthermore, MS patients can benefit from the use of electronic images by having their results transmitted to a more experienced neuro-ophthalmological center, receiving rapid treatment options in case of disease exacerbation and recurrence of ON.

Morales Dominguez et al. performed a study that attempted to validate a color gradation scale of the optic nerve photograph. In their study, three examiners analyzed and graded 150 photographs of optic discs from patients with glaucoma, non-glaucomatous optic neuropathies, and healthy ones and compared it with the computer-analyzed saturation and hue data of those images. Their scale (the Teheran–Morales) differentiated between vein red, normal, slight pallor, marked pallor, and waxy pallor. They concluded that there was a higher agreement between examiners regarding advanced glaucoma and non-glaucomatous optic neuropathies with pale discs and a substantially lower one in glaucomatous and healthy discs [17]. Our study design is similar, grading normal, light pallor, pallor, and strong pallor in patients with non-glaucomatous optic neuropathies, and it validates their conclusion of a strong agreement in evaluating optic disc atrophy in non-glaucomatous optic neuropathies. Furthermore, we utilize OCT thickness as a comparative method of analyzing disc atrophy, which increases the validity of our study.

Bambo et al. used a colorimetric software analysis devised for measuring optic disc pallor in MS patients using hemoglobin as a pigment of reference. Their analysis confirmed a greater temporal pallor of the optic disc in these patients, validating the property of the software to detect optic nerve pallor [19]. Our study differs by using a subjective grading from the examiners to evaluate optic disc pallor and OCT to correlate temporal optic disc neurodegeneration in a larger patient lot.

Smartphone fundus photography has been proven to be a cheap, portable, effective, and highly available method of evaluating posterior segment elements. These images can be analyzed to better understand disease activity and progression and observe modifications of the vascular architecture in systemic conditions. In addition, they can be easily accessed by telemedicine services in order to provide expert opinions from trained physicians. Moreover, the use of artificial intelligence may hasten this process and provide a quick analysis, highlighting the resulting modifications [20,21]. Patel et al. observed a 96% agreement between diagnoses of retinal pathologies identified on smartphone-based FP devices in the pediatric population, highlighting the potential of such devices to increase accessibility to ophthalmological care in communities with limited possibilities [22].

Wintergerst et al. performed a comparative study in order to observe the differences between undilated and dilated smartphone-based FP for optic nerve head evaluation. While their analysis focused on a different optic neuropathy, glaucoma, they used a similar quantification for optic disc pallor grading from pale to borderline and vital [23]. Our analysis used four steps, the difference resulting from the analysis of pallor from the temporal quadrant compared with other quadrants.

Gelfand et al. observed that pRNFL impairment is present in all MS patients, regardless of ON history, with greater severity in those with a long history of neurodegenerative activity. Eyes affected by ON exhibited a more advanced degree of pRNFL atrophy compared with NON eyes. Temporal quadrant involvement was predominant and characteristic of the MS population, as the small fibers of the parvocellular pathway are primarily located in this region. Their results also identified these changes following a clinical isolated syndrome, suggesting that neurodegeneration occurs early in the disease course and is independent of inflammatory activity [24]. These findings suggest that optic nerve pallor can appear in advanced cases regardless of ON history.

Retinal imaging modalities have given life to new dedicated tools that have led to an increase in the evidence of the prognostic value of such explorations in the study of cardiovascular and cerebrovascular diseases. The use of such software could provide insight into the pathogenic mechanism of neurodegenerative conditions, and, by focusing on machine learning protocols and automated programs, new ocular biomarkers could be identified that correlate not only with the progression of retinal disease activity [25] but with systemic pathologies as well [26]. The key advantage of these programs is their ability to continuously refine results by incorporating new data and parameters. Arian et al. demonstrated these capabilities through the analysis of OCT and infrared laser ophthalmoscopy (SLO) scans, noting an improvement in automated retinal lesion detection systems for multiple sclerosis [27]. Aghababei et al. confirmed these findings by utilizing a convoluting neural network to assess SLO scans in MS and healthy patients, observing promising results in the automated detection of MS lesions when applying image processing protocols [28].

Thompson et al. trained a deep learning algorithm that used fundus photographs and SD OCT data regarding Bruch membrane opening to predict neuro-retinal lesions in glaucomatous eyes. Their study provided a high diagnostic sensibility for assessing the loss of visual field in glaucoma [29]. In a different deep learning model, Yang et al. were successfully able to train an algorithm to discriminate between non-glaucomatous and glaucomatous optic neuropathies, with their design relying on color FPs [30].

New parameters such as retinal vascular and perfusion density measured through OCT-Angiography have benefited from many studies in recent years, attempting to establish correlations between modifications in the retinal vascular architecture and the inflammatory status during disease activity [31]. The inclusion of both structural and vascular OCT-A-derived parameters has led to an increase in the capacity for diagnosing MS eyes, regardless of their ON status [32]. Recent studies using OCT and widefield SLO imaging confirmed previous findings regarding the modification of retinal volume in MS patients, while offering new insights into changes in the retinal peripheral vasculature, suggesting the presence of a significant vascular component that contributes to retinal alterations in the pathological process of MS [33].

Animal study models using SLO provided data regarding leukocyte migration in MS-like experimental autoimmune encephalomyelitis, observing distinct movement patterns in the retinal vasculature contributing to the understanding of the leukocytic role in disease onset and progression [34]. Using laser speckle flowgraphy and OCT on animal models, researchers observed evidence of retinal hypoperfusion during the inflammatory process in ON similar to vascular changes in the central nervous system of MS patients [35].

By adding the optic nerve as part of the central nervous system, many previously underestimated patients with asymptomatic optic nerve damage could benefit from additional screening and diagnosis focused on exploring the intraocular part of the optic nerve [36]. While our analysis proved capable of discerning moderate and severe pallor as important markers for optic nerve degeneration, light pallor has been mistaken for normal, therefore enhancing the need for additional neuro-ophthalmological exploration.

In a retrospective study used to evaluate the indicators of poor prognosis in children with ON, Averseng-Peaureaux concluded that age over 10 years old, disc pallor, and previous positive MS diagnosis represent factors for poor recovery. While young age could be a limitation for in-depth ophthalmological investigations of the anterior optic path, evaluation of the optic disc and comparison with the other eye are still widely accessible, therefore justifying the continuous need for ophthalmoscopy in children with MS [37].

Due to the retrospective nature of this analysis, our study presents several limitations. These limitations include a low number of patients and a lack of correlation with clinical aspects such as visual acuity, contrast sensitivity or color vision, disease severity, disability status (EDSS), and disease duration. Examiner subjectivity could also be a potential source of errors. While, in our study, the examiners agreed on strong optic disc pallor, there were slight differences in other pallor-level gradings. This should be taken into consideration as different examiners might offer a subjective opinion that could negatively impact the analysis. Future studies regarding optic disc photography should focus on improving these aspects in order to limit sources of error and to bring additional evidence of the importance of optic disc pallor in evaluating everyday quality of life in MS patients.

While there are numerous investigative methods used for evaluating the posterior segment of the eye, fundoscopy and FP still remain the most universally available and commonly used techniques for screening retinal and optic disc lesions. Therefore, our study offers an insight into the accuracy of fundoscopy and FP in assessing optic disc degeneration and atrophy by a comparison with OCT-obtained pRNFL thickness, an established parameter for optic nerve degeneration. We believe our results to increase the value of FP in the evaluation of neuro-ophthalmological patients, especially as a basic screening step that could mark the way for the development of standardized objective deep learning algorithms that would remove any examiner biases.

## 5. Conclusions

Due to their universal availability, fundus ophthalmoscopy and photography are useful tools that can be used as an initial method of screening for optic nerve atrophy and evaluating the activity of the neurodegenerative process in MS patients. While FP is inferior to OCT in evaluating the progression of optic disc atrophy, our results provide evidence of the capacity of FPs evaluation to distinguish between normal and moderate or strong optic nerve atrophy using FP disc pallor grading.

With the proposed inclusion of the optic disc as the fifth part of the CNS, the optic nerve will require an increase in the accessibility and number of exploratory techniques in order to further the understanding of disease progression and increase patient quality of life, with FP examination having important diagnostic value in neuro-ophthalmological diseases, especially in low-resource conditions.

## Figures and Tables

**Table 1:** Subjective evaluation of optic disc pallor.

Temporal Quadrant Color	Pallor Grade
Intense orange	Normal
Light orange	Light pallor
Yellow	Pallor
White, waxy aspect	Strong pallor

**Table 2:** OCT temporal pRNFL thickness values based on pallor.

Grade	*n*	Mean (µm)	Std. Deviation (µm)	Minimum (µm)	Maximum (µm)
Normal	29	53.17	12.499	30	85
Light pallor	26	56.27	13.678	38	81
Pallor	24	42.75	11.349	22	60
Strong pallor	9	37.67	17.486	21	77

**Table 3:** OCT temporal thickness median analysis between optic disc fundoscopy scores.

Group Comparison	OCT Thickness Difference	Std. Error	*p*	Adjusted *p*
Strong pallor–Pallor	9.715	9.981	0.330	1.000
Strong pallor–Normal	28.933	9.744	0.003	0.018
Strong pallor–Light pallor	32.47	9.876	0.001	0.006
Pallor–Normal	19.218	7.047	0.006	0.038
Pallor–Light pallor	22.755	7.229	0.002	0.010
Normal–Light pallor	−3.537	6.897	0.608	1.000

## Data Availability

The original contributions presented in this study are included in the article. Further inquiries can be directed to the corresponding author.
